# Comparing transcriptional responses to Fusarium crown rot in wheat and barley identified an important relationship between disease resistance and drought tolerance

**DOI:** 10.1186/s12870-020-02818-1

**Published:** 2021-02-03

**Authors:** Z. Y. Su, J. J. Powell, S. Gao, M. Zhou, C. Liu

**Affiliations:** 1 CSIRO Agriculture and Food, St Lucia, Queensland 4067 Australia; 2 Tasmanian Institute of Agriculture, University of Tasmania, Prospect, TAS 7250 Australia; 3 School of Agriculture and Food Sciences, The University of Queensland, St Lucia, Brisbane 4072 Australia

**Keywords:** *Fusarium*, Wheat, Barley, Crown rot, Drought tolerance, RNA-seq

## Abstract

**Background:**

Fusarium crown rot (FCR) is a chronic disease in cereal production worldwide. The impact of this disease is highly environmentally dependant and significant yield losses occur mainly in drought-affected crops.

**Results:**

In the study reported here, we evaluated possible relationships between genes conferring FCR resistance and drought tolerance using two approaches. The first approach studied FCR induced differentially expressed genes (DEGs) targeting two barley and one wheat loci against a panel of genes curated from the literature based on known functions in drought tolerance. Of the 149 curated genes, 61.0% were responsive to FCR infection across the three loci. The second approach was a comparison of the global DEGs induced by FCR infection with the global transcriptomic responses under drought in wheat. This analysis found that approximately 48.0% of the DEGs detected one week following drought treatment and 74.4% of the DEGs detected three weeks following drought treatment were also differentially expressed between the susceptible and resistant isolines under FCR infection at one or more timepoints. As for the results from the first approach, the vast majority of common DEGs were downregulated under drought and expressed more highly in the resistant isoline than the sensitive isoline under FCR infection.

**Conclusions:**

Results from this study suggest that the resistant isoline in wheat was experiencing less drought stress, which could contribute to the stronger defence response than the sensitive isoline. However, most of the genes induced by drought stress in barley were more highly expressed in the susceptible isolines than the resistant isolines under infection, indicating that genes conferring drought tolerance and FCR resistance may interact differently between these two crop species. Nevertheless, the strong relationship between FCR resistance and drought responsiveness provides further evidence indicating the possibility to enhance FCR resistance by manipulating genes conferring drought tolerance.

**Supplementary Information:**

The online version contains supplementary material available at 10.1186/s12870-020-02818-1.

## Background

Fusarium crown rot (FCR), which can be caused by various *Fusarium* species with *F. pseudograminearum* being the dominant pathogen in most regions, is a chronic disease in wheat and barley production in semi-arid regions worldwide [1–3]. Previous studies showed that the disease could reduce grain yield by up to 35.0% in USA [4], 43.0% in Turkey [5] and 45.0% in Iran [6]. Based on surveys on production losses conducted a decade ago, FCR can bring about approximate AUD100 million loss annually in common wheat (*Triticum aestivum* L., genome AABBDD; 2n=6x=42) and barley (*Hordeum vulgare* L., genome HH, 2n=2x=14) production in Australia [7, 8].

Physical contact of stem bases or roots with stubble from infected plants from preceding years facilitates initial infection of *F. pseudograminearum* [9]. Seedling death does occur when the disease is severe [3], likely leading to yield loss in crop production. However, there are no reported studies investigating possible effects of seedling death on grain yield. Typical symptoms of the disease include browning of coleoptile, sub-crown internode, lower leaf sheaths and stem-base and root tissue during vegetative stages of plant growth. ‘White heads’ with shrivelled or no kernels are a common feature of FCR infected plants especially in wheat crops which suffered drought stress after flowering [1, 8].

FCR development under field conditions is pronounced under drought conditions, particularly post-anthesis [10]. It is also well documented that, although this disease occurs widely in cereal-growing regions worldwide, it causes serious grain yield loss mainly in semi-arid regions [4–6, 11, 12]. A histological study showed that, once the *Fusarium* pathogens get into the host plants, drought conditions enhance the proliferation and spread of pathogens [13]. Drought stress is also a critical step in encouraging severe FCR infection in the laboratory-based bioassay [14] which has been widely used in recent years in both common wheat [15–17] and barley [18–21]. However, it is not clear why drought stress enhances the severity of FCR infection and possible relationships between genes induced by FCR infection and drought tolerance have not been reported.

To facilitate the process of breeding FCR resistant varieties, significant efforts have been put into the identification of novel sources of resistance and detection of loci conferring FCR resistance. These efforts have resulted in the successful identification of quantitative trait loci (QTL) conferring FCR resistance in both wheat and barley [22]. Near isogenic lines (NILs) have been developed and used to validate putative loci identified from QTL mapping studies [18, 20, 23]. In the efforts of developing diagnostic markers for loci conferring FCR resistance, transcriptomic sequences from NILs targeting three of the FCR resistance loci (located on 4HL and 1HL in barley and on 3BL in wheat) were made available [15, 24, 25]. These available transcriptome sequences were used to study possible relationships between genes induced by FCR infection and drought tolerance based on two different approaches. Firstly, a selected panel of genes with known functions in drought tolerance was assessed against the transcriptome sequences from the NILs targeting each of the three loci conferring FCR resistance. We then further compared global DEGs from the 3BL RNA-seq data with global transcriptomic responses under drought in wheat. Results obtained from these assessments are reported in this publication.

## Results

### Molecular responses to crown rot were enriched for drought related processes in wheat but not in barley

**Fig. 1:**
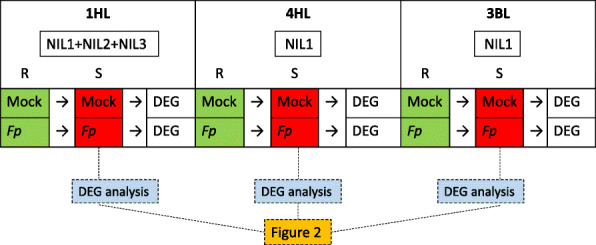
Diagram of the strategy for detecting differentially expressed genes (DEGs). Only comparisons of R^C^ and S^C^, and R^I^ and S^I^ were employed for the data analysis in this study. Data of each comparison from per targeted locus were classified into up- and down- regulated DEGs. Resistant and ‘S’ isolines from different NIL pairs were respectively merged for obtaining expression values by removing values with inconsistent symbols. Specifically, positive numbers will be retained when screening up-regulated DEGs but they will be removed when screening down-regulated ones

Comparison of DEGs between resistant (‘R’) and susceptible (‘S’) isolines following control-inoculation and *F. pseudograminearum*-inoculation (Supplementary Table 1) was conducted to determine whether DEGs which were shared among 1HL (barley), 4HL (barley) and 3BL (wheat) had significant enrichment for drought related processes and functions. Data from control (R^C^ and S^C^) and FCR-inoculated (R^I^ and S^I^) treatments were available across five pairs of the NILs for DEG analysis. They include three NIL pairs at one inoculation timepoint for 1HL, one NIL pair at two timepoints for 3BL, and one NIL pair at one timepoint for 4HL. To provide fairer comparison, isolines from different pairs and sampling timepoints were merged for each locus. Details of steps taken in the analysis were showed in Fig. 1 below.

**Fig. 2:**
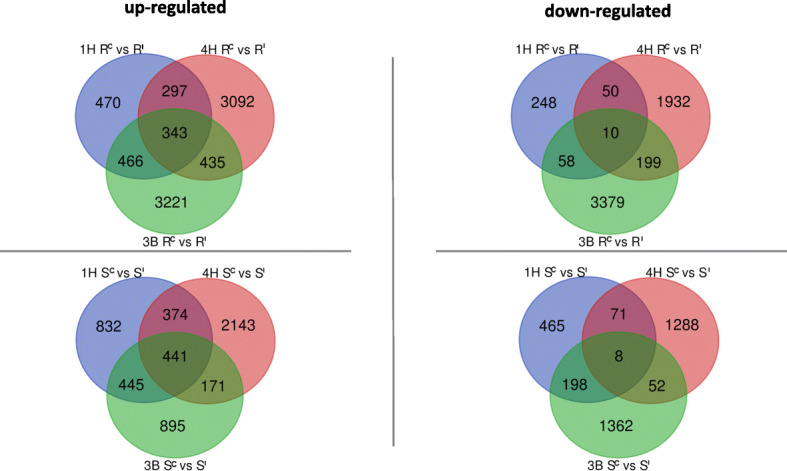
Number of unique and common DEGs between ‘R’ and ‘S’ isolines following control-inoculation and *F. pseudograminearum*-inoculation from three loci. ‘R’ and ‘S’ mean resistant and ‘S’ isolines, and ‘I’ and ‘C’ refer to *F. pseudograminearum* infection and control inoculation. The upper panel indicates common and unique genes up-regulated or down-regulated in the resistant isolines under infected conditions among 1HL, 4HL and 3BL NILs while the lower panel indicates common and unique up-regulated or down-regulated genes in the resistant isolines under control condition among NILs of the three targeted loci

A small degree of overlap in genes responding to infection were detected betweenthe three loci for up-regulated genes (Fig. 2). In total, 253 and 327 overlapping DEGs were identified in ‘R’ and ‘S’ isolines respectively under *F. pseudograminearum*-inoculation. However, few commonly down-regulated genes were observed among the three loci between ‘R’ and ‘S’ isolines with only 6 and 4 DEGs identified in ‘R’ and ‘S’ isolines respectively. The relatively small overlap between co-differentially expressed genes (both up-regulated and down-regulated) among the three loci indicates the existence of substantial differences in molecular response to infection, driven by differences in either the functions of the three different loci or between the two species.

GO term enrichment analysis was only performed on sets of up-regulated DEGs from changes following FCR infection in both the ‘R’ and ‘S’ isolines for each of the loci individually. Some drought associated GO terms were identified in 3BL R^C^ vs R^I^ (response to water deprivation and abscisic acid-activated signalling pathway) and 3BL S^C^ vs S^I^ (response to water deprivation and maintenance of seed dormancy by abscisic acid) while drought associated GO terms were not found to be enriched in 4HL or 1HL comparisons (Supplementary Table 2). To assess whether responses to infection within ‘R’ and ‘S’ isolines across all three loci were enriched for drought-related processes, GO term enrichment analysis was performed with the 253 and 327 overlapping up-regulated DEGs from R^C^ vs R^I^ and S^C^ vs S^I^ comparison. In total, 50 and 42 GO terms were obtained respectively. Most of the common GO terms from R^C^ vs R^I^ and S^C^ vs S^I^ comparison were related to pathogen defence associated processes; however, a close association with drought associated responses was not identified within the sets of common DEGs.

### Genes conferring drought tolerance responded to infection in both wheat and barley but only responded differently between isolines in 3BL and 1HL NILs

**Fig. 3:**
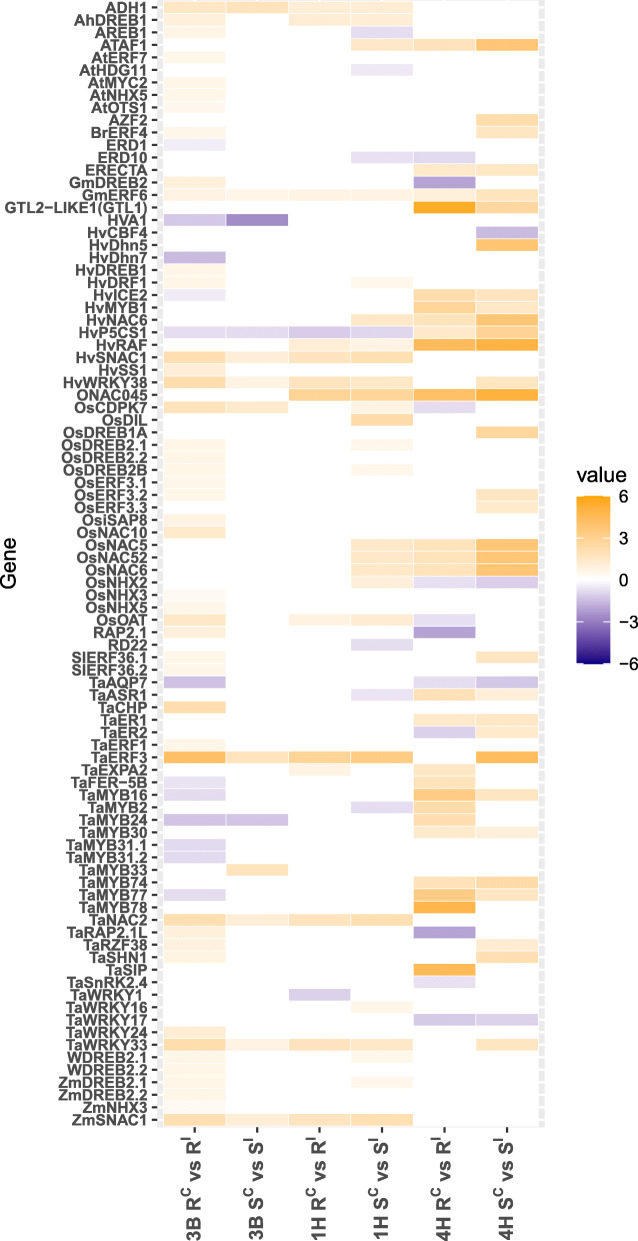
A heatmap showing fold-changes of drought related genes between resistant and susceptible isolines for the NILs targeting the three FCR loci. ‘R’ and ‘S’ indicate resistant and susceptible isolines, respectively, and ‘I’ and ‘C’ refer to *F. pseudograminearum* infection and control inoculation, respectively. Columns indicate fold-changes for different comparisons (combinations) while rows indicate changes for individual genes across the comparisons. The magnitude of fold-change is represented by colour intensity with yellow tones representing up-regulation and purple tones down-regulation

To assess the relationship between drought tolerance and FCR induced DEGs, a set of 149 genes related to drought tolerance were curated from the literature (Supplementary Table 3). Wheat and barley homologs for these drought genes were inferred from the Ensembl Plants database of global homologs [26] and changes to expression levels under infection were observed across the NILs. Interestingly, 91 of 149 drought tolerance related genes (61.0%) were differentially expressed in response to infection within one or more isolines (Fig. 3).

The majority of responsive drought tolerance genes were up-regulated under infection across NILs. For 4HL barley NILs, highly similar responses were observed between ‘R’ and ‘S’ isolines with 33 and 36 DEGs detected respectively. In contrast, 1HL susceptible isolines showed a much stronger drought response compared to ‘R’ isolines with 33 DEGs compared with 15 DEGs, perhaps indicating ‘S’ isolines were experiencing greater drought stress due to infection. The strongest difference observed was between 3BL isolines with a substantially heightened response in ‘R’ isolines (54 DEGs) compared to ‘S’ isolines (13 DEGs). These results indicate drought tolerance genes may form an important component of the response to crown rot infection in both wheat and barley in a resistance locus dependant manner and the 3BL resistance allele may be directly or indirectly regulating drought tolerance responses.

For commonly responsive genes across all comparisons, *GmERF6* and *HvP5CS1* were the mostly expressed genes in all of the comparisons among 1HL, 4HL and 3BL. *OsERF3*, a known gene negatively influenced drought tolerance in rice [27], represented up-regulation in ‘R’ isolines of 3BL and ‘S’ isolines of 4HL. Based on the enrichment of drought related terms in 3BL isolines alone and also the different response pattern to infection of drought tolerance related genes between 3BL isolines, it appeared that drought tolerance may be integral to the function of the 3BL resistance locus. Further exploration of the relationship between response to infection in 3BL NILs and response to drought based on previous RNA-seq data was undertaken to understand whether the overall response to infection in ‘R’ and ‘S’ isolines differed in drought-related processes.

### Global transcriptional responses to *F. pseudograminearum* infection and drought in wheat shows significant negative correlation

In order to assess the overlap between the transcriptomic response in the R isoline during infection with drought induced gene expression in an unbiased manner, we further compared global DEGs from the 3BL RNA-seq data with global wheat transcriptomic responses under drought. To this end, the pattern of transcriptional change within and between ‘R’ and ‘S’ isolines was compared with global drought induced transcriptional change within previously published RNA-seq dataset which observed gene expression differences between well-irrigated (control) and droughted wheat under field conditions [28]. To aid direct comparison with the 3BL RNA-seq dataset, this drought RNA-seq dataset was re-analysed using the same analysis pipeline, genome assembly and annotation to maintain technical consistency. Ma et al. [28] performed RNA-seq on leaf tissues from a drought tolerant wheat variety at different timepoints after irrigation (T4 = one-week post-irrigation and T6 = three weeks post-irrigation). In total, 296 and 834 significant DEGs responding to drought were detected at T4 and T6 respectively in our analysis (Supplementary Table 1).

**Fig. 4:**
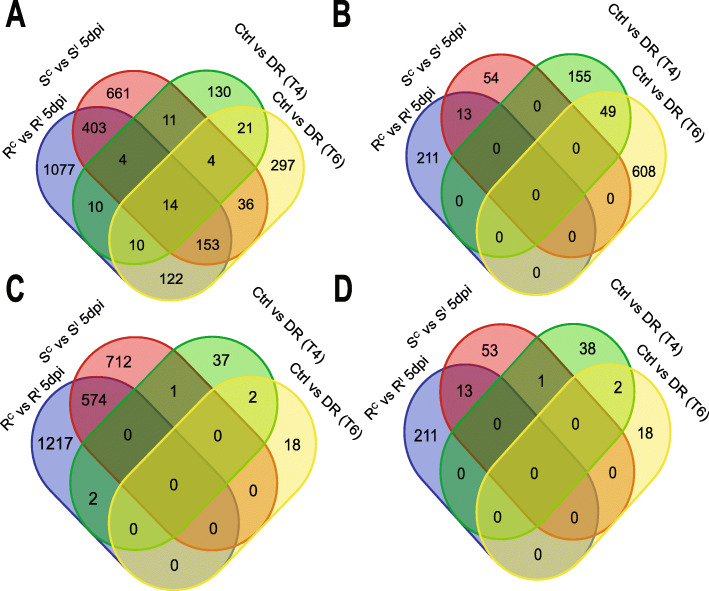
Venn diagrams displaying the overlaps between up-regulated and down-regulated DEGs identified in the comparison between resistant versus susceptible isolines targeting the 3BL locus with DEGs responsive to drought in wheat. Panel **a** displays the overlap between up-regulated RC vs R^I^ and S^C^ vs S^I^ DEGs at 5 dpi and Panel **b** displays down-regulated R^C^ vs R^I^ and S^C^ vs S^I^ DEGs at 5 dpi versus DEGs downregulated under drought (T4 and T6). Panel **c** displays the overlap between up-regulated S^I^ vs R^I^ DEGs (3dpi and 5dpi) and Panel **d** displays down-regulated S^I^ vs R^I^ DEGs versus DEGs up-regulated under drought (T4 and T6)

**Fig. 5:**
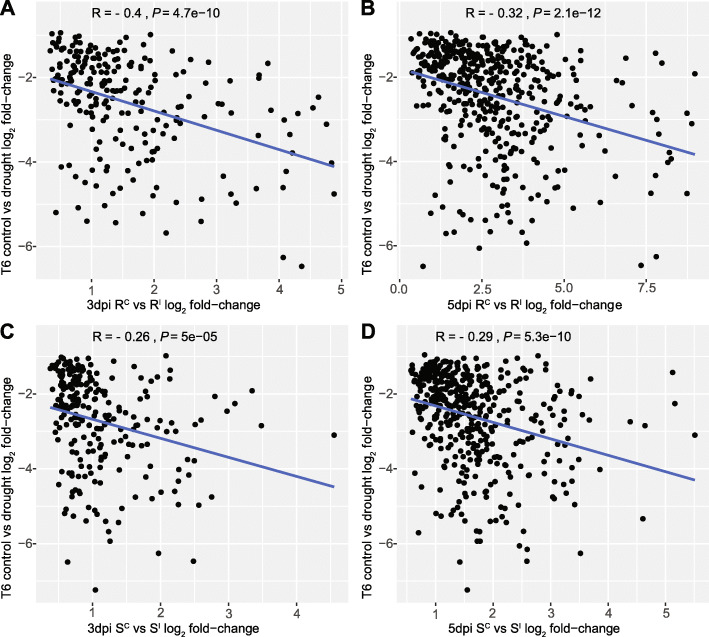
Scatterplots showing the correlation between DEG expression values for S^I^ vs R^I^ up-regulated genes versus genes down-regulated under drought. Panels **A** and **B** compare 3dpi and 5dpi R^C^ vs R^I^ up-regulated genes with drought T6 down-regulated genes. Panels **C** and **D** compare 5dpi S^C^ vs S^I^ up-regulated genes with drought T6 down-regulated genes. *r* values show the correlation co-efficient (Pearson) between DEG expression values. Axes display the log_2_ differential expression fold-changes during infection (x-axis) versus log_2_ differential expression fold-changes under drought (y-axis). Blue lines represent the line of best fit and shading shows the pointwise 95.0% confidence interval of the regression

Comparing drought responsive DEGs with *Fusarium* responsive genes within the ‘R’ and ‘S’ isolines indicated that the vast majority of genes responsive to both infection and drought were up-regulated under infection but down-regulated under drought with similar overlap proportion in both ‘R’ and ‘S’ isolines (17.0% and 16.0% respectively) (Fig. 4). Significant correlations were observed for both ‘R’ and ‘S’ isolines (3 dpi and 5 dpi) and T6 drought (Fig. 5). However, the correlations between ‘R’ isoline DEGs was stronger (r = − 0.4 for 3 dpi and r = − 0.32 for 5 dpi) compared to ‘S’ isoline DEGs (r = − 0.26 for 3 dpi and r = − 0.29 for 5dpi). Significant correlations were not observed between *Fusarium* induced DEGs in ‘R’ or ‘S’ isolines and T4 drought DEGs.

Given the weaker correlations observed for ‘S’ isolines DEGs compared with ‘R’ isoline DEGs, genes which were found to be expressed to different magnitudes between ‘R’ and ‘S’ isolines under infection were also compared with drought DEGs. Approximately 46.0% of T4 DEGs and 66.0% of T6 DEGs were also differentially expressed between S and R isolines under infection at one or more timepoint (Supplementary Figure 1). Interestingly, the vast majority of common DEGs were down-regulated under drought and expressed more highly in the ‘R’ isoline than the sensitive isoline under infection (Supplementary Figure 1 Panel A). To determine whether these common DEGs showed similar magnitudes of differential expression, genes expressed more highly in the ‘R’ isoline at each timepoint were selected and compared to genes which were up-regulated under drought conditions. Performing a pairwise correlation test (Pearson) revealed a moderately strong and highly significant negative correlation between each of the S^I^ vs R^I^ and drought comparisons with *r* values of − 0.43 and − 0.53 when comparing S^I^ vs R^I^ 3dpi DEG expression values with drought T4 and T6 DEG expression values respectively and *r* values of − 0.33 and − 0.39 when comparing S^I^ vs R^I^ 5dpi DEG expression values with drought T4 and T6 DEG expression values respectively (Supplementary Figure 2).

To explore which processes and functions were impacted by both FCR infection and drought, gene ontology enrichment analysis was performed using genes which were upregulated in 3BL ‘R’ isolines at 3 dpi and 5 dpi but down-regulated under drought stress at T6 (other comparisons did not contain enough common DEGs to allow for GO term enrichment analysis). This analysis revealed 199 and 110 enriched terms for 3 dpi and 5 dpi respectively (FDR *P* < 0.05; Bonferroni correction). Reducing these results to most specific terms, twenty-three and nineteen terms were identified for 3 dpi and 5 dpi respectively. Within the set of most specific terms, a large proportion of terms were associated with defence responses against fungal pathogens including defence response, production of anti-microbial metabolites and proteins with cinnamic acid biosynthetic process, L-phenylalanine catabolic process and chitin binding, defence phytohormones evinced by response to jasmonic acid and phenylalanine ammonia-lyase activity and also detoxification of a major FCR associated mycotoxin, deoxynivalenol with quercetin 7-O-glucosyltransferase activity enriched. Other general stress and drought stress related responses were also enriched included glutathione metabolic process, negative regulation of gibberellic acid mediated signalling pathway, hyperosmotic salinity and response negative regulation of abscisic acid-activated signalling pathway. Together these patterns infer that drought tolerance is enhanced within the 3BL ‘R’ isoline and that important defence responses implicated in response to *F. pseudograminearum* are both switched down during drought and more highly expressed in the ‘R’ compared to the ‘S’ isoline during FCR infection.

## Discussion

FCR is a chronic disease in many cereal-growing regions worldwide but the disease causes severe yield loss mainly in drought-affected crops. Based on the available transcriptome sequences from NILs targeting three different loci conferring FCR resistance in wheat and barley, we investigated possible relationships between genes induced by FCR and drought using two different approaches. Results from these assessments are highly consistent in that more than half of drought related genes were detected among DEGs induced by FCR infection. This study represents the first comparative study of global transcriptional responses to drought with transcriptional responses related to FCR resistance in these crop species. Observed patterns indicated a strong inverse relationship between FCR resistance and drought responsiveness in wheat with most commonly responsive genes downregulated during drought but expressed more highly in the ‘R’ isoline. Enrichment analysis indicated that many of these genes play roles in known defences elicited against *F. pseudograminearum* such as deoxynivalenol detoxification [29–31] and induced systemic resistance signalling mediated by the phytohormones, jasmonate and salicylic acid [29, 30, 32] but also within abscisic acid signalling, a known pathway which plays a role in both coordinating drought tolerance mechanisms in wheat [33, 34] and resistance to *Fusarium* pathogens [35, 36]. The resistance locus could be mediating resistance through two distinct mechanisms: directly limiting the ingress, spread and proliferation of the pathogen inside the plant within the ‘R’ isoline or the resistance locus may indirectly impact resistance by enhancing tolerance to drought conditions allowing the ‘R’ isoline to elicit a stronger defence response owing to greater access to water resources. These findings also indicate that many induced defences against *F. pseudograminearum* may have reduced effectiveness within drought affected wheat crops due to their concerted down-regulation during drought responses, particularly within wheat varieties with greater sensitivity to drought.

However, genes induced by drought stress were predominantly up-regulated in the susceptible isolines under FCR infection for both assessed loci in barley. These results indicate that genetic networks controlling FCR resistance and drought tolerance likely significantly overlap but genes conferring drought tolerance may affect FCR resistance differently between these two different crop species. The different relationship between genes induced by FCR infection and drought is another feature differing wheat and barley in regarding to FCR resistance. Results from previous studies showed that, compared to wheat, barley seedlings accumulated *Fusarium* pathogens much faster at each of the stages following FCR inoculation [37]. One of the possible factors contributing to the differences between these two crop species is their difference in ploidy level. It is generally assumed that an polyploid genome offers a buffering effects when facing abiotic as well as biotic challenge, allowing it to adapt to wide ranges of envirionments [38]. However, compared with other cereal species, the diploid barley has an extremely wide geographic range and it is particularly able to adapt to diverse environments varying greatly in water availablity [39]. This unique feature of barley could be a reason why the relationship between genes induced by FCR infection and drought stress in this species seems to be different from that in common wheat.

Importantly, drought stress played no role in obtaining the three sets of transcriptome data from the NILs targeting each of the three loci assessed in this study. Results from a histological study showed that drought-stress prolongs the initial infection and enhances the proliferation and spread of *Fusarium* pathogens after the initial infection phase [13]. To promote disease development, FCR inoculated seedlings well watered in the first few days following inoculation and then drought stress is imposed [14]. However, the transcriptome sequences used in this study were all obtained from seedlings of 3 or 5-day post inoculation when they had not been exposed to drought stress [15, 24, 25].

Previous findings indicate drought stress enhances the proliferation and spread of *Fusarium* pathogens after the initial infection phase) [13]. From the results of the current study, the strong relationship between FCR resistance and drought responsiveness also points to the feasibility of enhancing FCR resistance by manipulating genes conferring drought tolerance. However, it is well known that genes conferring drought tolerance may have different mechanisms, express in different tissues and each of them may only be effective at a certain stage of plant development [40, 41]. Further, loci conferring FCR resistance have been located on different locations in the genomes of both wheat and barley [22] and it is not unreasonable to speculate that genes conferring FCR resistance may have different mechanisms. Thus, it would not be surprising that the effectiveness of drought genes on FCR resistance may vary and that different genes conferring FCR resistance may response differently to different genes conferring drought tolerance.

The interaction between drought and FCR resistance has important implications both for the identification and functional study of genes involved in FCR resistance as well as for efforts to improve this trait within breeding programs. Future efforts to clarify the relationship between drought and resistance for implicated loci should phenotype NILs for drought tolerance within controlled environments either using chemically simulated droughting with polyethylene glycol solution [42] or controlled soil water content saturation/deficit irrigation schedules [43]. If clear differences in drought tolerance phenotypes are observed, further global transcriptomic studies in which isolines are treated with various combinations of drought stress and FCR infection would identify shared and distinct networks of transcriptional response between the two stresses. Differences in transcriptional or translational regulation [44, 45] of biosynthetic pathways may also infer differential accumulation of metabolites which could be validated and used as biomarkers for screening germplasm for combined drought tolerance and FCR resistance [43, 46, 47]. The strong interaction between effective resistance and drought tolerance may also necessitate that phenotyping germplasm to identify novel sources of resistance should also incorporate an element of drought stress into the screening procedure as per the method used to identify the resistance sources and their associated NILs utilized in this study [14]. In this way, there can be more confidence that the resistance alleles underpinning these phenotypes will provide tracible resistance within the environments that FCR typically inflicts constraint on grain yield. Our findings may also indicate that effective screening strategies should target germplasm which has improved drought tolerance or at least has been selected within environments in which water availability is a significant constraint.

## Conclusions

FCR is a chronic disease in all semi-arid regions worldwide but severe yield loss from this disease occurs mainly in drought-affected crops and drought stress is known to enhance the proliferation and spread of pathogens within infected plants. By examining genes introduced by FCR infection and drought stress, we detected strong relationship at gene expression level between these two characteristics. Our results suggest the possibility of exploiting some of the genes conferring drought tolerance to enhance FCR resistance although the effectiveness of these genes may differ between the two crop species studied. To make such an approach more effective, those genes conferring drought tolerance at different stages of plant development should be targeted.

## Methods

To assess possible relationship between FCR resistance and drought tolerance, transcriptomic sequences from multiple NILs targeting three loci conferring FCR resistance were used in the study reported here. One of these loci is located on the long arm of chromosome 3B in common wheat [15]. The other two are barley loci, one on the long arm of chromosome 4H [18] and the other on the long arm of chromosome 1H [25]. The transcriptomic sequences from the different NILs in each of the three studies were obtained from plants which were either control inoculated with water or inoculated with a single isolate of *Fusarium pseudograminearum* (CS3096). In addition, transcriptomic sequences were also obtained from a previous study observing drought responses in wheat [28]. Two timepoints from this study were selected for re-analysis: ‘T4’ comprised of leaf tissue sampled at one-week post-irrigation and ‘T6’ comprised of leaf tissue sampled at three weeks post-irrigation representing mild to moderate drought stress within plants undergoing vegetative growth (control samples were taken from well-irrigated plants at the same growth stage within the same trial).

All of the transcriptome sequences used in this study were retrieved from the National Centre for Biotechnology Information (NCBI) (https://www.ncbi.nlm.nih.gov/). Accession numbers for the three sets of transcriptome sequences induced by FCR infection were PRJNA541021 (for the locus on 1HL), PRJNA392021 (4HL) and SRP048912 (3BL), respectively. The accession number for the transcriptome sequences from the study on drought tolerance was SRP102636.

The methods used for analysing the transcriptome data were those described in detail by Habib et al. [24]. Basically, FastQC v0.11.2 (http://www.bioinformatics.babraham.ac.uk/projects/fastqc/) was applied to check the acceptable scores for PHRED. SolexaQA ++ v3.1.3 (http://solexaqa.sourceforge.net/) was used to trim and filter the raw RNA reads with the minimum PHRED quality score of 30 and minimum final read length of 70 bp. TopHat2 v2.2.13 was used for aligning filtered reads to the reference genome of barley (based on the genotype Morex) and common wheat (based on the genotype Chinese Spring). Quantification of transcript abundance in samples was assessed with Cufflinks (version 2.02) as described by Roberts et al. [48]. Cufflinks (http://cufflinks.cbcb.umd.edu/) was used to produce a sample-wise annotation based on transcripts identified from aligned RNA-seq reads [49]. All sample assemblies were merged with the high-confidence transcriptome annotation of ‘Morex’ for barley and ‘Chinese Spring’ for wheat, respectively, using Cuffmerge from the Cufflinks tool package. CuffDiff was used to calculate fragments per kilobase of exon per million mapped read (FPKM) values and perform pairwise comparisons between genotype/treatment combinations to identify DEGs. Fold change (in log_2_ scale) was calculated as fold change = log_2_ (*FPKM*_*A*_/ *FPKM*_*B*_).

The combinations of genotype-treatments were analysed for pairwise comparisons in two ways: different treatments for the same isoline (R^C^ vs R^I^ and S^C^ vs S^I^) and the same treatment between the ‘R’ and ‘S’ isolines (R^C^ vs S^C^ and R^I^ vs S^I^). ‘C’ and ‘I’ stand for control treatment (inoculated with water) and *F. pseudograminearum*-inoculation. Significant DEGs from each comparison were determined by setting an adjusted *P* value (Bonferroni correction) threshold of ≤ 0.05 and log_2_ expression fold change of ≥ 1 or ≤ − 1 or ‘inf’ or ‘-inf’ (in one condition FPKM value is zero and the other is not).

DEGs were identified following *F. pseudograminearum*- and control-inoculation from both the ‘R’ and ‘S’ isolines for NILs targeting each of the loci. Global wheat and barley homologs were obtained from Ensembl Plants (http://plants.ensembl.org) using the BioMart tool [26, 50]. Venn diagrams were generated to compare similarities and differences using the method described in the website http://bioinformatics.psb.ugent.be/webtools/Venn. To assess genes related to drought tolerance, their transcript names were matched with DEGs from each of the three FCR loci to obtain specific values for comparison.

A total of 149 genes conferring drought tolerance were curated from various plant species based on literature searches [51, 52]. The homologs of these genes in wheat and barley were identified and they were all reported as key genes involved in drought response. Nucleotide sequence for each of these genes was obtained from NCBI.

Global wheat and barley coding sequences were annotated with Blast2GO (https://www.blast2go.com/) using standard parameters following the method described by Conesa et al. [53]. DEGs induced by FCR within ‘R’ and ‘S’ isolines for all three loci and DEGs induced by both drought and FCR between the ‘R’ and ‘S’ isolines of the NILs targeting the 3BL locus were tested separately for GO enrichment using the Fisher’s exact test enrichment module to identify significantly enriched GO terms (*P* value < 0.05; Fisher’s exact test) with ‘most specific terms’ filter applied within Blast2GO as described in Habib et al. [24].

## Supplementary Information

**Additional file 1: Supplementary Figure 1.** Venn diagrams displaying the overlaps between up-regulated and down-regulated DEGs identified in the comparison between resistant versus susceptible isolines targeting the 3BL locus with DEGs responsive to drought in wheat. Panel A displays the overlap between up-regulated S^I^ vs R^I^ DEGs (3dpi and 5dpi) and Panel B displays down-regulated S^I^ vs R^I^ DEGs versus DEGs downregulated under drought (T4 and T6). Panel C displays the overlap between up-regulated S^I^ vs R^I^ DEGs (3dpi and 5dpi) and Panel D displays down-regulated S^I^ vs R^I^ DEGs versus DEGs up-regulated under drought (T4 and T6).

**Additional file 2: Supplementary Figure 2.** Scatterplots showing the correlation between DEG expression values for S^I^ vs R^I^ up-regulated genes versus genes down-regulated under drought. Panels A and C compare 3dpi S^I^ vs R^I^ up-regulated genes with drought T4 and drought T6 down-regulated genes respectively. Panels B and D compare 5dpi S^I^ vs R^I^ up-regulated genes with drought T4 and drought T6 down-regulated genes, respectively. *r* values show the correlation co-efficient (Pearson) between DEG expression values. Axes display the log_2_ differential expression fold-changes for S^I^ vs R^I^ (x-axis) versus log_2_ differential expression fold-changes under drought (y-axis). Blue lines represent the line of best fit and shading shows the pointwise 95.0% confidence interval of the regression.

**Additional file 3: Supplementary Table 1.** Global list of significant DEGs for 3BL, 4HL and 1HL NILs from *Fusarium* infection RNA-seq studies and also DEGS from the drought response RNA-seq study.

**Additional file 4: Supplementary Table 2.** Gene Ontology enrichment analysis outputs for DEGs from various NIL/treatment comparisons.

**Additional file 5: Supplementary Table 3.** Details of the 149 drought tolerance associated genes curated from the literature for the targeted comparison of shared drought and crown rot transcriptional responses.

## Data Availability

The datasets analysed during the current study are available in the the National Centre for Biotechnology Information (NCBI) (https://www.ncbi.nlm.nih.gov/). Accession numbers for the three sets of transcriptome sequences induced by FCR infection were PRJNA541021 (for the locus on 1HL), PRJNA392021 (4HL) and SRP048912 (3BL), respectively. The accession number for the transcriptome sequences from the study on drought tolerance was SRP102636.
